# Antibiotic tolerance due to restriction of cAMP-Crp regulation by mannitol, a non-glucose-family PTS carbon source

**DOI:** 10.1128/msphere.00772-24

**Published:** 2024-11-20

**Authors:** Weiwei Zhu, Miaomiao Chen, Xue Zhang, Jie Su, Xinyang Zhang, Yuejuan Nong, Bowen Wang, Weihong Guo, Yunxin Xue, Dai Wang, Yiqun Liao, Jianjun Niu, Yuzhi Hong, Karl Drlica, Xilin Zhao

**Affiliations:** 1 State Key Laboratory of Vaccines for Infectious Diseases, Xiang-An Biomedicine Laboratory, Department of Laboratory Medicine, School of Public Health, Xiamen University, Xiamen, Fujian Province, China; 2 State Key Laboratory of Molecular Vaccinology and Molecular Diagnostics, Department of Laboratory Medicine, School of Public Health, Xiamen University, Xiamen, Fujian Province, China; 3 Center of Clinical Laboratory, Zhongshan Hospital, School of Medicine, Xiamen University, Xiamen, Fujian Province, China; 4 MOE Key Laboratory of Geriatric Diseases and Immunology, Suzhou Key Laboratory of Pathogen Bioscience and Anti-infective Medicine, Institute of Molecular Enzymology, School of Life Sciences, Soochow University, Suzhou, Fujian Province, China; 5 Public Health Research Institute, New Jersey Medical School, Rutgers Biomedical and Health Sciences, Rutgers University, Newark, New Jersey, USA; 6 Department of Microbiology, Biochemistry & Molecular Genetics, New Jersey Medical School, Rutgers Biomedical and Health Sciences, Rutgers University, Newark, New Jersey, USA; University of Nebraska Medical Center College of Medicine, Omaha, Nebraska, USA

**Keywords:** antibiotic tolerance, mannitol, mannose, sorbitol, cAMP-Crp, *Escherichia coli*, ROS, *ptsI*, EIIA^Glc^

## Abstract

Enzyme-IIA (EIIA^Glc^, Crr) of the phosphotransferase system (PTS) connects the uptake of glucose-family sugars to the cAMP-Crp regulatory cascade; phosphorylated EIIA^Glc^ enhances cAMP-Crp activity, which then contributes to the antibiotic-mediated accumulation of reactive oxygen species (ROS) and cell death. Defects in PTS cause antibiotic and disinfectant tolerance. We report that mannitol, a carbon source whose uptake does not use EIIA^Glc^, reduces antibiotic-mediated killing of *Escherichia coli* without affecting antibiotic minimal inhibitory concentration. Thus, mannitol promotes antibiotic tolerance. The tolerance pathway was defined by the loss of ciprofloxacin lethality from the deletion of *ptsI* (first gene in PTS), *mtlA* (mannitol-specific Enzyme-II), *cyaA* (cAMP synthase), and *crp* (cAMP receptor protein) but not *crr* (EIIA^Glc^). A crp* mutant, which encodes a constitutively active Crp that bypasses the need for cAMP activation, also decreased mannitol-mediated antibiotic tolerance, as did exogenous cAMP. Thus, inhibition of antibiotic lethality by mannitol involves both PTS-mediated mannitol uptake and suppression of cAMP-Crp action, independent of EIIA^Glc^. Mannitol suppressed the downstream antibiotic-mediated transcription of genes involved in NADH production and cellular respiration, expression of a superoxide reporter gene (*soxS*), and accumulation of antibiotic-mediated ROS. Similar phenomena were observed with mannose and sorbitol, demonstrating that non-glucose PTS carbon sources can cause antibiotic tolerance by a novel path that reduces the ROS-promoting activity of cAMP-Crp. The work emphasizes that antibiotic tolerance, which contributes to disease relapse and the need for prolonged antibiotic treatment, can result from commonly consumed carbohydrates. This finding, plus mutations that interfere specifically with antibiotic lethality, makes tolerance a high probability event.

**IMPORTANCE:**

Bacterial tolerance constitutes a significant threat to anti-infective therapy and potentially to the use of disinfectants. Deficiency mutations that reduce glucose uptake, central carbon metabolism, and cellular respiration confer antibiotic/disinfectant tolerance by reducing the accumulation of reactive metabolites, such as reactive oxygen species. We identified novel environmental generators of tolerance by showing that non-glucose carbohydrates, such as mannitol, mannose, and sorbitol, generate tolerance to multiple antibiotic classes. Finding that these sugars inhibit a universal, stress-mediated death pathway emphasizes the potential danger of compounds that block the lethal response to severe stress. Immediate practical importance derives from mannitol being a popular food sweetener, a treatment for glaucoma, and a dehydrating agent for treating cerebral edema, including cases caused by bacterial infection: antibiotic tolerance could contra-indicate the use of mannitol and related carbohydrates during antibiotic treatment. Overall, the work shows that the presence of sugars must be considered during antimicrobial and perhaps disinfectant use.

## OBSERVATION

**Fig 1:**
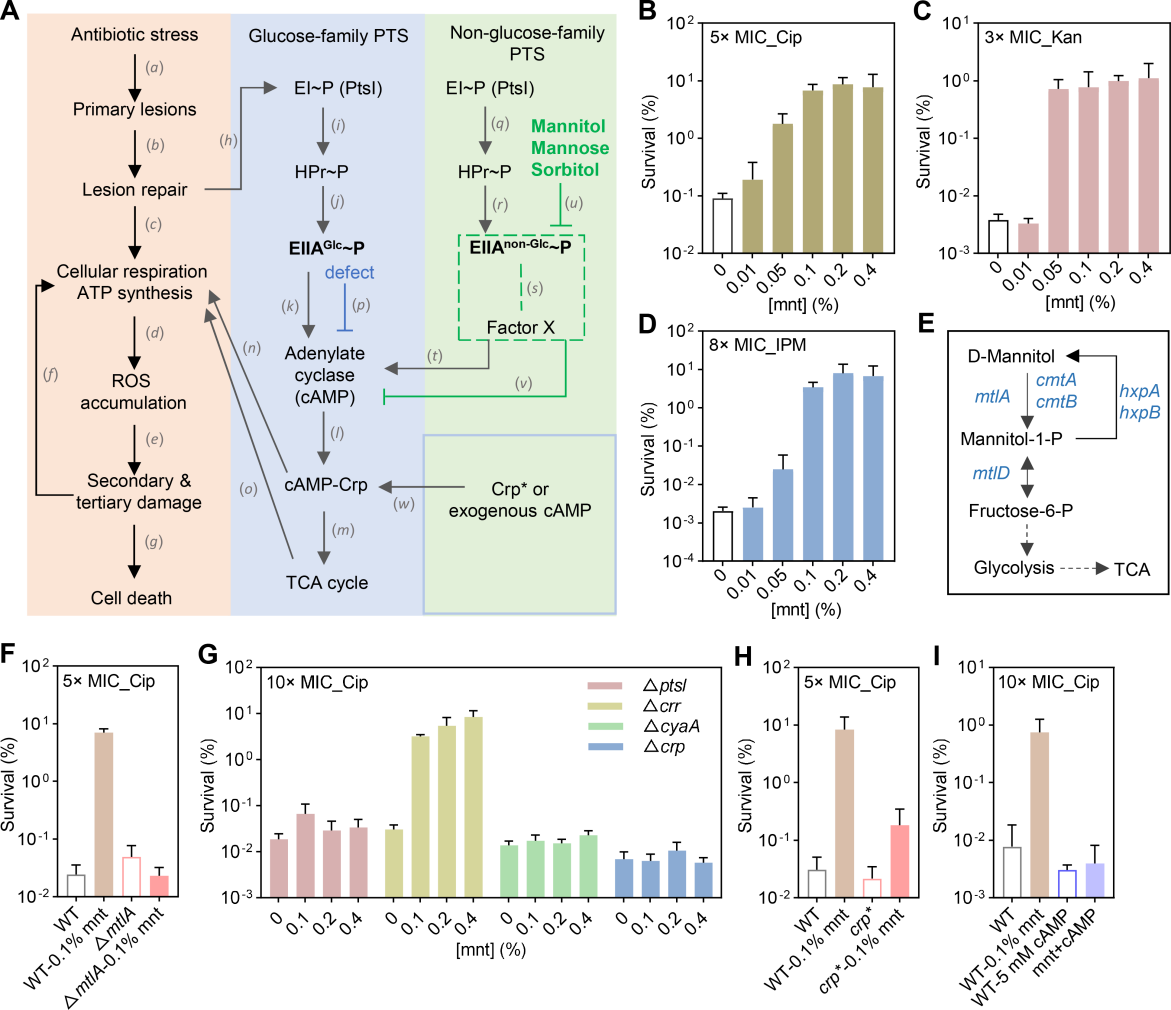
cAMP-Crp involvement in mannitol-mediated antibiotic tolerance. (**A**) Scheme showing a PTS-cAMP-Crp-mediated antibiotic-mediated death pathway. Primary lesions at specific targets (*a*), caused by lethal antibiotics, increase demand for ATP to repair damage (*b*), leading to elevated ATP synthesis (*c*) via respiration that produces ROS as a byproduct (*d*) that causes oxidative damage to macromolecules (*e*), further increasing demand for ATP (*f*) and additional accumulation of ROS (*d*). Oxidative damage (*d–f*) results in cell death (*g*). Substrates for ATP synthesis can be obtained by stimulating the PTS-cAMP-Crp cascade (*h*). EI and HPr (*i*) phosphorylate glucose-specific EIIA^Glc^ (*j*), which then stimulates adenylate cyclase to increase cAMP levels (*k*). cAMP combines with Crp (*l*) to promote the TCA cycle (*m*), cellular respiration (*n* and *o*), ROS accumulation, and cell death (*d–g*). Defects in enzyme I (PtsI), Hpr, or EIIA^Glc^ cause pan-tolerance to antibiotics and disinfectants by reducing cAMP levels (*p*). EI and HPr (*q*) phosphorylate non-glucose-family EIIA (*r*), resulting in elevated adenylate cyclase activity and cAMP promoted by factor X (*s*). That stimulates cAMP-Crp regulatory activity (*t*) and subsequent cell death. Uptake of mannitol, mannose, or sorbitol decreases levels of phosphorylated EIIA^non-Glc^ (*u*), thereby limiting the activation of adenylate cyclase by factor X (*v*). Crp* and exogenous cAMP bypass mannitol action (*w*). (**B–D**) Tolerance to commonly used antibiotic classes. Survival of *E. coli* BW25113 (strain 60) pre-incubated with the indicated concentrations of mannitol for 15 min before treatment with ciprofloxacin for 2 h, kanamycin for 2 h, or imipenem for 6 h. (**E**) Genes involved in mannitol metabolism. Dashed lines indicate that intermediate processes have been omitted. (**F**) Effect of Δ*mtlA* on mannitol tolerance to ciprofloxacin. Δ*mtlA* mutant (strain 1929) and wild-type cells (strain 60) were treated with 0% or 0.1% mannitol for 15 min; then, ciprofloxacin was added for 2 h before survival was determined. (**G**) Effect of deficiencies in PTS-cAMP-Crp cascade genes on mannitol-mediated tolerance to ciprofloxacin. Δ*ptsI* mutant (strain 990), Δ*crr* mutant (strain 979), Δ*cyaA* mutant (strain 695), and Δ*crp* mutant (strain 1063) were pre-incubated with the indicated concentrations of mannitol for 15 min after which ciprofloxacin was added for 2 h. (**H and I**) Effect of crp* mutant or exogenous cAMP on mannitol-mediated protection from ciprofloxacin lethality. crp* mutant (strain 1196) and wild type (strain 60) were pre-incubated with the indicated concentrations of mannitol (**H**) or cAMP (**I**) for 15 min, after which ciprofloxacin was added for 2 h. At least three biological replicates were performed; error bars represent standard deviations. Cip, ciprofloxacin; Kan, kanamycin; IPM, imipenem; and mnt, mannitol.

Studies with *Escherichia coli* describe a death process (Fig. 1A) in which lethal antibiotics and disinfectants cause lesions in bacteria that stimulate carbohydrate metabolism, the TCA cycle, cellular respiration, and production of lethal reactive metabolites, such as reactive oxygen species (ROS). The result is bacterial death (1–3). This type of stress response can involve the phosphotransferase system (PTS) enzyme-I (EI, PtsI) and HPr proteins that phosphorylate the glucose-family-specific enzyme-II (EIIA^Glc^, Crr). Phosphorylated EIIA^Glc^ then stimulates adenylate cyclase (CyaA) to produce cAMP, which combines with the cAMP receptor protein (Crp) to promote the TCA cycle, cellular respiration, and subsequent cell death (Fig. 1A) (3). Deficiency mutations define the death pathway by creating antibiotic/disinfectant tolerance (loss of lethal activity while retaining inhibition of growth). Although the importance of the PTS-cAMP-Crp cascade in promoting antibiotic lethality is clear (3, 4), still unknown is the effect of non-glucose-family PTS carbon sources, such as mannitol, that rely on phosphorylated MtlA (EIIABC) for uptake rather than EIIA^Glc^ (Fig. 1A) (5).

When we examined how mannitol affects antibiotic lethality, we were surprised to find that this sugar increases *E. coli* survival to ciprofloxacin, kanamycin, and imipenem by at least 100-fold (Fig. 1B through D). Since mannitol had no effect on either *E. coli* culture growth (Fig. S1A) or the minimal inhibitory concentration (MIC) for the antimicrobials (Table S1), its effect is specific to lethal action: it causes antibiotic tolerance.

Two mannitol-specific uptake PTS EII exist, MtlA and CmtAB (5, 6) (Fig. 1E). Tests using ciprofloxacin showed that in the absence of *mtlA*, but not *cmtA*, mannitol failed to increase *E. coli* survival (Fig. 1F; Fig. S2A); tolerance was also eliminated by a *ptsI* deficiency (Fig. 1G; Table S2). Thus, both PtsI and MtlA are in the mannitol tolerance pathway, presumably due to their role in uptake. Deletion of downstream genes *mtlD*, *hxpA*, or *hxpB* blocked further conversion of mannitol-1-phosphate (Fig. 1E) (7), although with these mutants, mannitol still mediated tolerance to ciprofloxacin (Fig. S2B through D). As expected, mannitol did not affect ciprofloxacin MIC with the mutants examined (Table S3).

To determine whether mannitol increases *E. coli* survival in cells with mutations in genes downstream from the PTS-mediated phosphorelay (3), we examined *cyaA* and *crp* deficiencies. When we treated mutant cultures with mannitol and lethal concentrations of ciprofloxacin (10× MIC), deletion of *cyaA* or *crp* eliminated mannitol-mediated ciprofloxacin tolerance, indicating that these genes are in the mannitol tolerance pathway (Fig. 1G). Deletion of *crr* had no effect (Fig. 1G; EIIA^Glc^, encoded by *crr*, normally promotes CyaA activity by phosphorylation). The crp* mutation (constitutive for cAMP-Crp activity) (8, 9) and exogenously added cAMP each reduced mannitol-mediated protection from ciprofloxacin lethality (Fig. 1H and I; Table S4). Overall, the data indicate that mannitol triggers tolerance by reducing cAMP levels, thereby blocking the function of cAMP-Crp independently of EIIA^Glc^.

**Fig 2:**
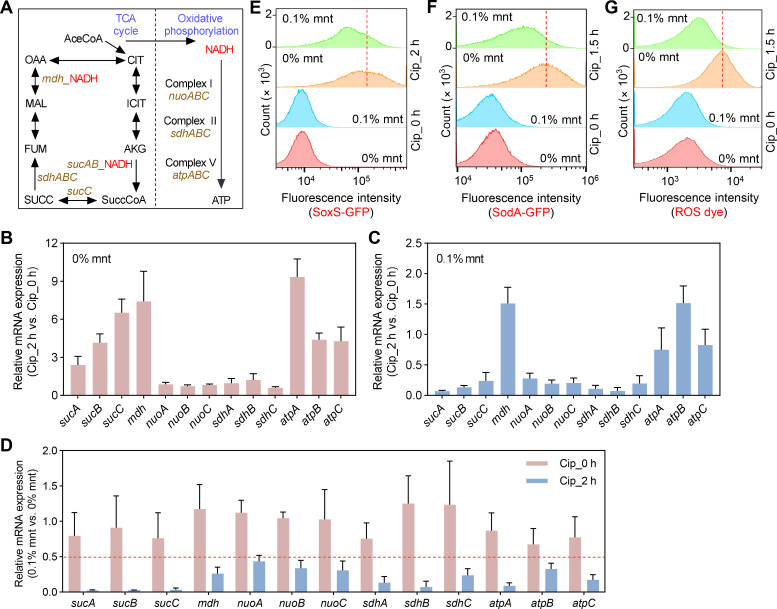
Inhibition of TCA cycle, cellular respiration, and ROS levels associated with mannitol-mediated antibiotic tolerance. (**A**) Scheme showing genes in TCA cycle and oxidative phosphorylation. PYR, pyruvate; AceCoA, acetyl-Coenzyme A; CIT, citrate; ICIT, isocitrate; AKG, α-ketoglutarate; SuccCoA, succinyl-CoA; SUCC, succinate; FUM, fumarate; MAL, malate; and OAA, oxaloacetate. (**B–D**) Relative transcript levels of the indicated genes involved in TCA cycle and oxidative phosphorylation at 0% or 0.1% mannitol incubated for 15 min before 5× MIC ciprofloxacin treatment for 0 and 2 h. (**E**) SoxS expression associated with ciprofloxacin and mannitol treatment. Fluorescence of a *soxS-gfp* fusion (strain 1944) was monitored by flow cytometry following pre-incubation with the indicated concentrations of mannitol for 15 min before ciprofloxacin (5× MIC) was added to exponentially growing cultures for the indicated times. (**F**) SodA expression associated with ciprofloxacin and mannitol treatment. Conditions were as in panel C except for the use of *sodA-gfp* (strain 1943). (**G**) Intracellular ROS levels. Wild type (strain 60) was treated with the indicated concentrations of ciprofloxacin and mannitol following treatment of cells with carboxy-H2DCFDA. ROS was monitored by flow cytometry. In RT-qPCR experiments, at least three biological replicates were performed; error bars represent standard deviations. In panels E–G, flow cytometry analysis was performed in three biological replicates, with the same trend in the results (see Fig. S3 for two additional repetitions). In panel D, the red dotted line indicates relative expression below 0.5, indicating a difference of more than twofold. In panels E–G, the red dashed line indicates the peak fluorescence intensity after ciprofloxacin treatment of cells in the absence of mannitol. Cip, ciprofloxacin; mnt, mannitol.

Suppression of central carbon metabolism and cellular respiration by blocking cAMP-Crp activity confers tolerance to a variety of stresses that include multiple antibiotics and disinfectants (3). To determine whether mannitol has the same effect, we examined the transcription of *mdh* and *sucABC*, genes involved in NADH production in the TCA cycle, and *nuoABC*, *sdhABC*, and *atpABC*, which encode proteins of the cellular respiratory complex (Fig. 2A).

In the absence of mannitol, ciprofloxacin resulted in a significant increase in the transcript levels of *sucABC*, *mdh,* and *atpABC* but exhibited little effect on the expression of *nuoABC* and *sdhABC* (Fig. 2B). When 0.1% mannitol was added, ciprofloxacin-stimulated transcription of *mdh*, *sucABC*, and *atpABC* was drastically suppressed (Fig. 2C). Indeed, the mannitol-ciprofloxacin combination decreased the transcript levels of all genes tested (e.g., relative expression level below 0.5) (Fig. 2D), including *nuoABC* and *sdhABC,* whose expression was not affected by ciprofloxacin alone (Fig. 2B). In the absence of antibiotic stress, mannitol had little effect on the expression of these genes (Fig. 2D). Thus, the suppression of transcription for these genes by mannitol specifically occurs during ciprofloxacin exposure. Mannitol, via suppression of cAMP-Crp, downregulates genes that contribute to ciprofloxacin-induced expression of respiration-related functions (*mdh* [10], *sucABC*, and *sdhABC* [11, 12]).

Inhibition of respiration-related gene expression was expected to reduce the accumulation of superoxide that accompanies respiration. Indeed, mannitol reduced the level of superoxide, as indicated by lower SoxS-GFP intensity detected with mannitol-treated cells (Fig. 2E; Fig. S3A) (SoxS is induced by superoxide [13]). The level of superoxide dismutase, represented by SodA (14), was also reduced by mannitol during ciprofloxacin-mediated stress (Fig. 2F; Fig. S3B). Bacterial intracellular ROS species are diverse, as superoxide is converted to hydrogen peroxide, which then forms hydroxyl radical via the Fenton reaction (15, 16). As a measure of diverse ROS, we treated cells with carboxy-H2DCFDA, a dye that fluoresces when oxidized (17). We found by flow cytometry that ciprofloxacin treatment increases the ROS signal and that mannitol (0.1%) reduces it (Fig. 2G; Fig. S3C). These data fit with the tolerance phenotype of mannitol treatment.

Two other non-EIIA^Glc^ sugars, mannose and sorbitol (5), behaved like mannitol in creating tolerance, as they failed to affect *E. coli* growth (Fig. S1B and C) or the MIC of several antibiotics (Table S1). Mannose caused tolerance to ciprofloxacin, kanamycin, and imipenem (Fig. S4); sorbitol-mediated tolerance was observed for ciprofloxacin and imipenem but not for kanamycin (Fig. S5). Understanding the difference between mannitol and sorbitol for kanamycin tolerance requires additional work. Mutations in *ptsI*, *cyaA*, and *crp* but not *crr* also eliminated mannose- and sorbitol-mediated antibiotic tolerance (Fig. S6). We conclude that mannose and sorbitol mediate antibiotic tolerance, as seen with mannitol, by restricting the cAMP-Crp regulatory activity that drives ROS-mediated death in an EIIA^Glc^-independent manner.

While the results described above solidify a role for cAMP-Crp in promoting bacterial death (3, 18–20), exactly how cAMP levels are regulated by mannitol requires further investigation (Fig. 1A, box formed by green dashed lines). Mannitol did not affect the transcript levels of *cyaA* and *crp* during ciprofloxacin stress (Fig. S7), nor did it mediate antibiotic tolerance by stimulating cAMP hydrolysis (Fig. S8): mannitol-mediated tolerance remained after deletion of the probable cAMP hydrolases CpdA and DosP (19) (Fig. S8). Thus, mannitol may not affect cAMP-Crp regulatory activity through known pathways. One possibility is that an uncharacterized factor, designated X, promotes adenylate cyclase activity and cAMP production (21, 22). The uptake of non-glucose PTS carbon sources, such as mannitol, mannose, and sorbitol, may inhibit the enhancement of adenylyl cyclase activity by Factor X. This would lead to low levels of cAMP and low cAMP-Crp activity, reducing downstream activity of the TCA cycle, respiration, the accumulation of ROS, and death. Crp* (constitutive cAMP-Crp activity) and adding exogenous cAMP can bypass the protective action of mannitol by directly rendering cAMP-Crp hyperactive (Fig. 1A) (3).

The potential for tolerance induced by mannitol in the diet or as a therapeutic can be mitigated by pausing mannitol consumption during treatment with lethal antibiotics. For example, in mannitol-assisted intracranial surgery, patient plasma mannitol concentration is maintained at levels expected to confer antibiotic tolerance (23) (Fig. 1B through D). A more general issue is the addition of a sugar-based mechanism to the mutational mechanisms that generate tolerance in bacteria by interfering with a major ROS-mediated death pathway (a different route to tolerance is seen when high levels of glucose mediate tolerance by stimulating fructose phosphate metabolism that inhibits an ROS surge from antibiotic stress [24]). Thus, bacteria likely have many ways to achieve tolerance to antibiotics and disinfectants, making tolerance a high-probability event. Since some compounds to which bacteria exhibit tolerance are used by the human immune system to kill bacterial pathogens (3), the generation of tolerance could be clinically quite important, because the immune system is likely to be crucial for clearing infection, even during antibiotic treatment (25). High throughput surveillance methods are now needed to assess the relationship between human activities, such as the massive use of disinfectants, and the prevalence of tolerance.

## Data Availability

Data supporting the findings of this study are shown in the main text and supplemental material.

## SUPPLEMENTAL MATERIAL

Supplemental Material msphere.00772-24-s0001.pdf Supplemental text, Fig. S1-S8, and Tables S1-S6.

ASM does not own the copyrights to Supplemental Material that may be linked to, or accessed through, an article. The authors have granted ASM a non-exclusive, world-wide license to publish the Supplemental Material files. Please contact the corresponding author directly for reuse.
