# Supplementary material for: Antibiotic tolerance due to restriction of cAMP-Crp regulation by mannitol, a non-glucose-family PTS carbon source
Source: mSphere. 2024 Nov 20;9(12):e00772-24. doi: 10.1128/msphere.00772-24 (PMC11656735; doi:10.1128/msphere.00772-24)
Supplement: Supplemental Material — Supplemental text, Fig. S1-S8, and Tables S1-S6. [file msphere.00772-24-s0001.pdf]

Supplementary Information

Antibiotic tolerance due to restriction of cAMP-Crp regulation by mannitol, a non-glucose-family PTS carbon source

Weiwei Zhu[a], Miaomiao Chen[a], Xue Zhang[a], Jie Su[a], Xinyang Zhang[a], Yuejuan Nong[a], Bowen Wang[a], Weihong Guo[a], Yunxin Xue[a], Dai Wang[a], Yiqun Liao[a], Jianjun Niu[b], Yuzhi Hong[c], Karl Drlica[d], Xilin Zhao[a,]*

[a] State Key Laboratory of Vaccines for Infectious Diseases, Xiang-An Biomedicine Laboratory & State Key Laboratory of Molecular Vaccinology and Molecular Diagnostics, Department of Laboratory Medicine, School of Public Health, Xiamen University, 4221-117 South Xiang-An Road, Xiang-An District, Xiamen, Fujian Province, 361102, China.

[b] Center of Clinical Laboratory, Zhongshan Hospital, School of Medicine, Xiamen University, 209 South Hubin Road, Siming District, Xiamen, Fujian Province 361004, China.

[c] MOE Key Laboratory of Geriatric Diseases and Immunology, Suzhou Key Laboratory of Pathogen Bioscience and Anti-infective Medicine, Institute of Molecular Enzymology, School of Life Sciences, Soochow University, 199 Ren-Ai Road, Suzhou, Jiangsu Province 215123, China

[d] Public Health Research Institute and Department of Microbiology, Biochemistry & Molecular Genetics, New Jersey Medical School, Rutgers Biomedical and Health Sciences, Rutgers University, 225 Warren Street, Newark, NJ 07103, USA.

* Address correspondence to: Xilin Zhao (zhaox5@xmu.edu.cn)

Table of Contents

Supplementary Materials and Methods

S1. Bacterial strains, culture, and reagents.
S2. Susceptibility determination.
S3. Bacterial killing assays.
S4. Construction of strains.
S5. Flow cytometry analysis.
S6. Total RNA extraction and cDNA synthesis.
S7. RT-qPCR.
S8. Statistical Analyses.

Supplementary Figures

Fig. S1. Effect of mannitol, mannose, or sorbitol on the growth of *E. coli*.
Fig. S2. Effect of deficiencies in various mannitol metabolic pathways on mannitol-mediated protection from ciprofloxacin lethality.
Fig. S3. Suppression of ROS levels associated with mannitol-mediated antibiotic tolerance.
Fig. S4. Effect of mannose on antibiotic-mediated killing of *E. coli*.
Fig. S5. Effect of sorbitol on antibiotic-mediated killing of *E. coli*.
Fig. S6. Effect of deficiencies in PTS-cAMP-Crp cascade on mannose, or sorbitol-mediated ciprofloxacin tolerance.
Fig. S7. Effect of the presence or absence of mannitol on *cyaA* and *crp* transcript levels during ciprofloxacin stress.
Fig. S8. Effect of cAMP hydrolases CpdA and DosP deficiency on mannitol-mediated antibiotic tolerance.

Supplementary Tables

Table S1. MIC of different antibiotics against strain BW25113 after addition of mannitol, mannose, or sorbitol.
Table S2. MIC of ciprofloxacin against Δ*ptsI*, Δ*crr*, Δ*cyaA and* Δ*crp* mutant strains after addition of mannitol, mannose, or sorbitol.
Table S3. MIC of ciprofloxacin against various *E. coli* mutants in the presence/absence of mannitol.
Table S4. MIC of ciprofloxacin against strain BW25113 after addition of mannitol or/and cAMP.
Table S5. Bacterial strains and plasmids used in the study.
Table S6. Primers used in the study.

Supplementary References

Supplementary Materials and Methods

S1. Bacterial strains, culture, and reagents

E. coli K-12 strains in the study are listed in Supplementary Table S5. The Keio Collection strains were verified by PCR using primers listed in Supplementary Table S6. All bacteria were cultured in Luria-Bertani (LB) medium at 37°C, with shaking at 200 rpm. Ciprofloxacin was purchased from Sigma-Aldrich Corp. (St. Louis, MO, USA). Kanamycin, mannitol, mannose, sorbitol, and H_2O_2 were purchased from Sangon Biotech Inc. (Shanghai, China). Imipenem was obtained from the Xiamen University Affiliated Zhongshan Hospital (Xiamen, China) pharmacy inventory. Carboxy-H2DCFDA was obtained from Thermo Fisher Scientific Corp. (Waltham, MA, USA).

S2. Susceptibility determination

MIC was determined by a 2-fold broth dilution method. Exponentially growing cultures were diluted to 10^5 cells/mL (mannitol, mannose, or sorbitol were added to the diluent at various concentrations as needed), mixed with various amounts of antimicrobial, and incubated at 37°C for 16 h. MICs were the lowest antibacterial concentration that allowed no visible turbidity increase.

S3. Bacterial killing assays

Overnight bacterial cultures were diluted 100-fold and re-grown to OD_{600} = 0.25~0.3; mannitol, mannose, or sorbitol were added 15 min before antimicrobials. At various times, aliquots were taken and subjected to 10-fold serial dilution in 0.9% saline. The diluted samples (10 μL) were spotted in triplicate on LB agar and incubated for 24 h for visual colony-forming unit (CFU) determination. Percent survival was determined relative to an untreated culture sampled at the time of antimicrobial addition.

S4. Construction of strains

A CRISPR-based allelic exchange was used to insert the *gfp* gene into the genome. Specifically, the *sodA*::*Kan*^{R} strain deleted the kanamycin resistance gene in the presence of plasmid pCP20, leaving the FRT (flippase recombinase target) sequence (*sodA*::FRT) recognizable by the pTargetF-FRT-expressing sgRNA. *E. coli* strain BW25113 was used as a template to amplify the *sodA* gene (without a stop codon) containing the upstream homology arm, and to amplify the downstream homology fragment of the *sodA* gene. The *gfp* gene fragment was amplified from the pAJR70 plasmid. The above PCR products were further ligated using overlap PCR to get the "upstream homology arm-*sodA*-*gfp*-downstream homology arm" recombinant fragment, which was used to replace the Cas9-cleaved FRT sequence in the *sodA*::FRT strain to obtain the BW25113 SodA-GFP strain. The *cpdA-dosP* double deletion strain was also constructed using the CRISPR method. The primers involved are listed in Supplementary Table S6.

S5. Flow cytometry analysis

Bacterial cultures grown to OD_{600} = 0.25~0.3 were treated with 5x MIC ciprofloxacin for the times in the figures. When appropriate, mannitol was added to cultures 15 min before ciprofloxacin treatment. To measure the total intracellular ROS levels, Carboxy-H2DCFDA, at a final concentration of 10 µM, was added to BW25113 cultures 15 min before ciprofloxacin treatment. For the characterization of intracellular superoxide levels, the *soxS-gfp* fusion expression plasmid was transformed into the BW25113 strain. The intensity of GFP fluorescence expressed in the genome in fusion with the *sodA* gene was used to indicate the expression levels of SodA. Aliquots taken at various times were washed twice with pre-chilled 1 ml phosphate-buffered saline, and then analyzed by flow cytometry (CytoFLEX, Beckman Coulter Inc., Beverly, MA, USA). All test samples recorded 10^5 cells under the condition of 525/40 nm band pass filter (FITC channel), and the generated FCS file was used for FlowJo-X analysis.

S6. Total RNA extraction and cDNA synthesis

Exponentially growing cultures of wild-type *E. coli* were treated with 5x MIC ciprofloxacin in the absence/presence of 0.1% mannitol for 0 min or 120 min. Samples were centrifuged (12,000 *g*, 4 ºC, 2 min) to collect cells. Subsequently, total RNA was extracted using an RNA extraction kit (TransGen Biotech Co., Beijing, China) following the manufacturer's protocol. Total RNA was reverse-transcribed using random primers, and single-stranded cDNAs were synthesized according to protocols of the All-in-One 5× RT MasterMix kit (Applied Biological Materials, Richmond, Canada), and the resulting cDNA samples were stored at −80°C.

S7. RT-qPCR

2× Universal SYBR Green Fast qPCR Mix (Abclonal, Woburn, MA USA) was utilized for the amplification of targeted PCR products using primers listed in Supplementary Table S6 using a qTOWER^3 G instrument (Analytik Jena AG, Jena, German). Detailed procedures were as follows: one cycle at 95°C for 3 min, then 40 cycles at 95°C for 5 s followed by 60°C for 30 s. The fluorescence value of 16S rRNA was used as an internal standard to calculate the relative expression of targeted genes using the 2(-DDCt) method (1).

S8. Statistical Analyses

All experiments were performed independently at least three times. Killing data were presented as mean ± SD.

Supplementary Figures

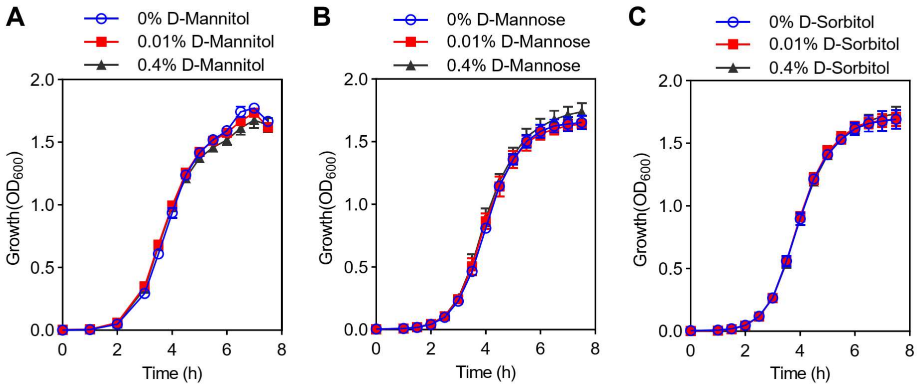

Fig. S1. Effect of mannitol, mannose, or sorbitol on the growth of *E. coli*. Overnight wild-type (strain 60) cultures were diluted 1000-fold and re-grown in LB medium. Mannitol (**A**), mannose (**B**), or sorbitol (**C**) was added to the diluted culture at the indicate concentrations. The optical density of the cultures was measured at the indicated times. At least three biological replicates were performed and error bars represent standard deviations.

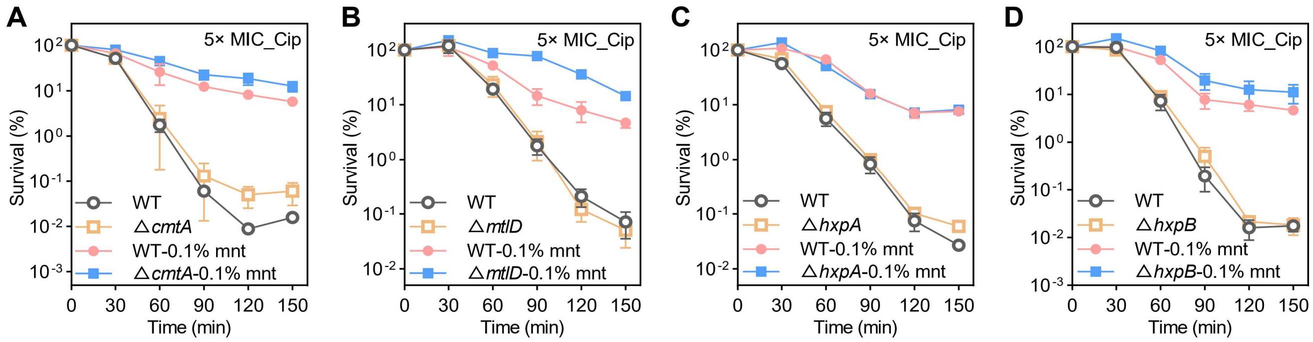

Fig. S2. Effect of deficiencies in various mannitol metabolic pathways on mannitol-mediated protection from ciprofloxacin lethality. Exponentially growing cultures (OD_{600}=0.25~0.3 in Luria-Bertani medium) of Δ*cmtA* mutant (strain 1931) (**A**), Δ*mtlD* mutant (strain 1936) (**B**), Δ*hxpA* mutant (strain 1934) (**C**), Δ*hxpB* mutant (strain 1935) (**D**), and wild-type (strain 60) strains were treated with 0% or 0.1% mannitol for 15 min before ciprofloxacin (5× MIC) was added to treat bacteria for the indicated times. At least three biological replicates were performed and error bars represent standard deviations. Abbreviations: mnt, mannitol; Cip, ciprofloxacin.

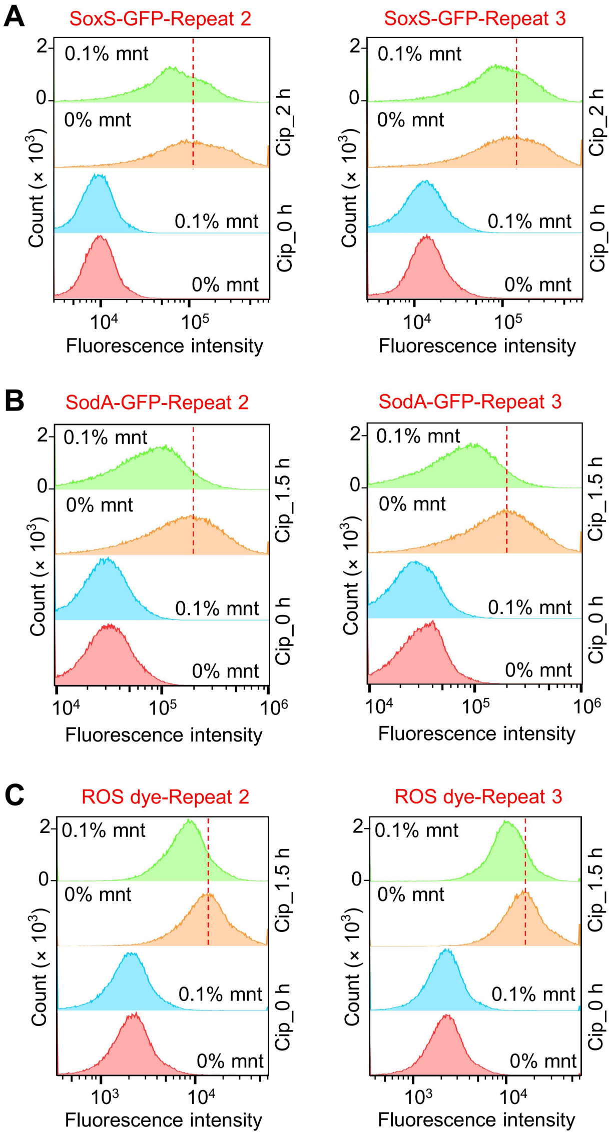

Fig. S3. Suppression of ROS levels associated with mannitol-mediated antibiotic tolerance. Repeats of Fig. 2E-G. (**A**) SoxS expression associated with ciprofloxacin and mannitol treatment. Fluorescence of a SoxS-GFP fusion (strain 1944) was monitored by flow cytometry following pre-incubation with the indicated concentrations of mannitol for 15 min before ciprofloxacin (5 × MIC) was added to exponentially growing cultures for the indicated times. (**B**) SodA expression associated with ciprofloxacin and mannitol treatment. Conditions were as in panel C except for use of SodA-GFP (strain 1943). (**C**) Intracellular ROS levels. Wild-type (strain 60) was treated with the indicated concentrations of ciprofloxacin and mannitol following treatment of cells with carboxy-H2DCFDA. ROS was monitored by flow cytometry. The red dashed line indicates the peak fluorescence intensity after ciprofloxacin treatment of cells in the absence of mannitol. Abbreviations: Cip, ciprofloxacin; mnt, mannitol.

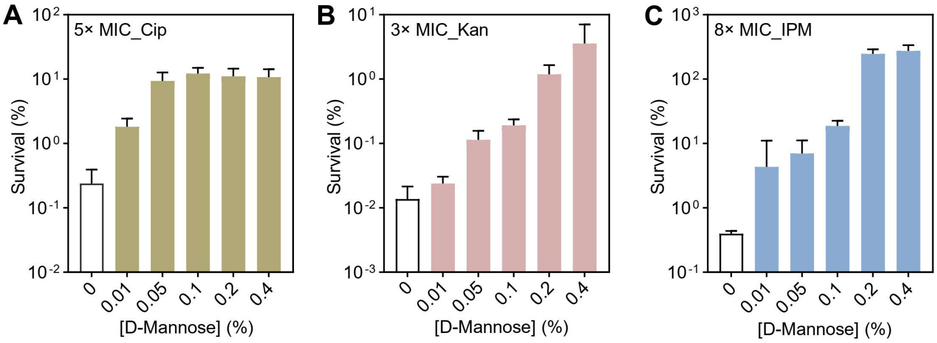

Fig. S4. Effect of mannose on antibiotic-mediated killing of *E. coli*. Survival of *E. coli* BW25113 (strain 60) pre-incubated with the indicated concentrations of mannose for 15 min before treatment with 5× MIC ciprofloxacin for 2 h (**A**), 3× MIC kanamycin for 2 h (**B**) or 8× MIC imipenem for 6 h (**C**). All experiments were performed in Luria-Bertani medium, and bacterial cultures were grown to exponential phase (OD_{600}=0.25~0.3) for antibiotic treatment. At least three biological replicates were performed; error bars represent standard deviations. Abbreviations: Cip, ciprofloxacin; Kan, kanamycin; IPM, imipenem.

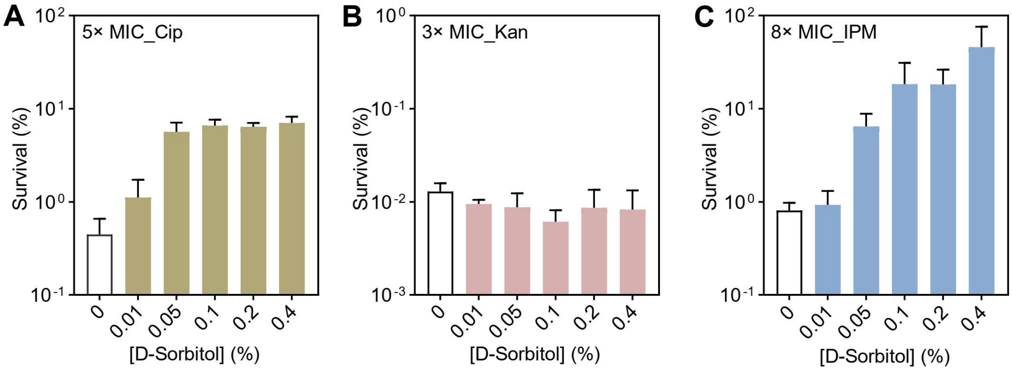

Fig. S5. Effect of sorbitol on antibiotic-mediated killing of *E. coli*. Survival of *E. coli* BW25113 (strain 60) pre-incubated with the indicated concentrations of sorbitol for 15 min and then treatment with 5× MIC ciprofloxacin for 2 h (**A**), 3× MIC kanamycin for 2 h (**B**) or 8× MIC imipenem for 6 h (**C**). All experiments were performed in the Luria-Bertani medium, and bacterial cultures were grown to the exponential phase (OD_{600}=0.25~0.3) for antibiotic treatment. At least three biological replicates were performed and error bars represent standard deviations. Abbreviations: Cip, ciprofloxacin; Kan, kanamycin; IPM, imipenem.

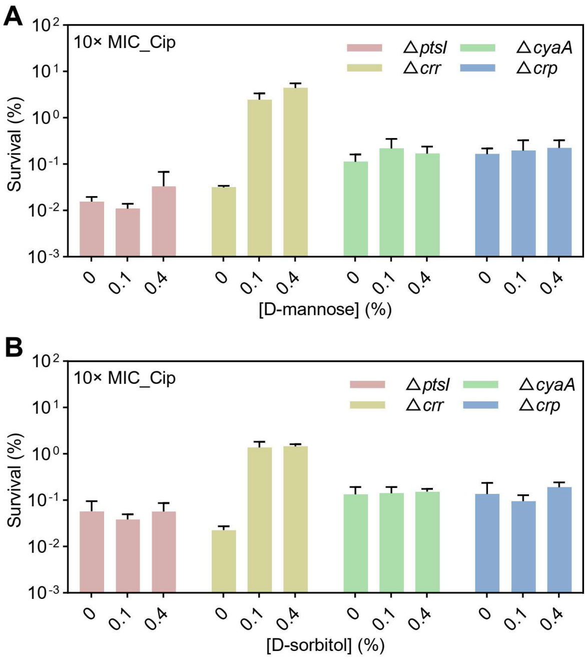

Fig. S6. Effect of deficiencies in PTS-cAMP-Crp cascade on mannose- or sorbitol-mediated ciprofloxacin tolerance. Exponentially growing cultures (OD_{600}=0.25~0.3 in Luria-Bertani medium) of *E. coli* strains were pre-incubated with 0%, 0.1% or 0.4% mannose (**A**) or sorbitol (**B**) for 15 min after which ciprofloxacin (10× MIC) was added for 2 h. At least three biological replicates were performed and error bars represent standard deviations. Abbreviations: Cip, ciprofloxacin. Δ*ptsI*, strain 990; Δ*crr*, strain 979; Δ*cyaA*, strain 695; Δ*crp*, strain 1063.

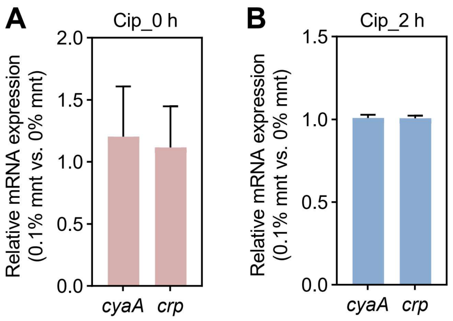

Fig. S7. Effect of the presence or absence of mannitol on *cyaA* and *crp* transcript levels during ciprofloxacin stress. Relative transcript levels of the indicated genes involved in TCA cycle and oxidative phosphorylation at 0% or 0.1% mannitol incubated for 15 min before 5× MIC ciprofloxacin treatment for 0 h and 2 h. At least three biological replicates were performed; error bars represent standard deviations. Abbreviations: Cip, ciprofloxacin; mnt, mannitol.

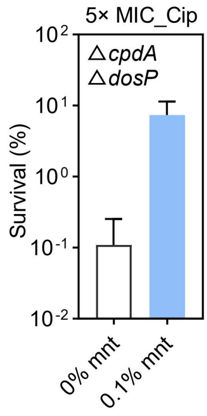

Fig. S8. Effect of cAMP hydrolases CpdA and DosP deficiency on mannitol-mediated antibiotic tolerance. Exponentially growing cultures (OD_{600}=0.25~0.3) of △*cpdA*△*dosP* (strain 1066) strain was treated with indicated concentrations of mannitol for 15 min and then 5× MIC ciprofloxacin was added for 2 h. At least three biological replicates were performed and error bars represent standard deviations. Abbreviations: Cip, ciprofloxacin; mnt, mannitol.

Supplementary Tables

Table S1. MIC of different antibiotics against strain BW25113 (strain 60) after addition of mannitol, mannose or sorbitol.

	Drugs MIC (mg/L)		
Additives	Cip	Kan	IPM
None	0.02	4	0.25
0.01% D-Mannitol	0.02	4	0.25
0.4% D-Mannitol	0.02	4	0.25
0.01% D-Mannose	0.02	4	0.25
0.4% D-Mannose	0.02	4	0.25
0.01% D-Sorbitol	0.02	4	0.25
0.4% D-Sorbitol	0.02	4	0.25

Table S2. MIC of ciprofloxacin against Δ*ptsI*, Δ*crr*, Δ*cyaA and* Δ*crp* mutant strains after addition of mannitol, mannose or sorbitol.

Additives concentration (w/v)	Strain/MIC (mg/L)			
	Δ*ptsI* (strain 990)	Δ*crr* (strain 979)	Δ*cyaA* (strain 695)	Δ*crp* (strain 1063)
0% mannitol	0.02	0.02	0.02	0.02
0.1% mannitol	0.02	0.02	0.02	0.02
0.4% mannitol	0.02	0.02	0.02	0.02
0% mannose	0.02	0.02	0.02	0.02
0.1% mannose	0.02	0.02	0.02	0.02
0.4% mannose	0.02	0.02	0.02	0.02
0% sorbitol	0.02	0.02	0.02	0.02
0.1% sorbitol	0.02	0.02	0.02	0.02
0.4% sorbitol	0.02	0.02	0.02	0.02

Table S3. MIC of ciprofloxacin against various *E. coli* mutants in the presence/absence of mannitol.

Mannitol concentration (w/v)	Strain/MIC (mg/L)					
	WT	Δ*mltA*	Δ*cmtA*	Δ*mltD*	Δ*hxpA*	Δ*hxpB*
0%	0.02	0.02	0.02	0.02	0.02	0.02
0.1%	0.02	0.02	0.02	0.02	0.02	0.02
	*crp**	Δ*cpdA*Δ*dosP*				
0%	0.015	0.02				
0.1%	0.015	0.02				

The strain numbers are shown in Table S5.

Table S4. MIC of ciprofloxacin against strain BW25113 (strain 60) after addition of mannitol or/and cAMP.

Chemicals	Ciprofloxacin MIC (mg/L)
None	0.02
0.1% mannitol	0.02
5 mM cAMP	0.01
0.1% mannitol+5 mM cAMP	0.01

Table S5. Bacterial strains and plasmids used in the study.

No.	Strain	Relevant Genotype	Source/reference
60	BW25113	*E. coli* wild-type *rrnB*3 Δ*lacZ*4787 *hsdR*514 Δ(*araBAD*)567 Δ(*rhaBAD*)568 *rph-1*	(2)
1929	Δ*mltA*	BW25113 Δ*mltA*::*kan*^{R}, Keio Collection, JW2784	(2)
1931	Δ*cmtA*	BW25113 Δ*cmtA*::*kan*^{R}, Keio Collection, JW2900	(2)
1936	Δ*mltD*	BW25113 Δ*mltD*::*kan*^{R}, Keio Collection, JW5018	(2)
1934	Δ*hxpA*	BW25113 Δ*hxpA*::*kan*^{R}, Keio Collection, JW5376	(2)
1935	Δ*hxpB*	BW25113 Δ*hxpB*::*kan*^{R}, Keio Collection, JW1716	(2)
695	Δ*cyaA*	BW25113 Δ*cyaA*::*kan*^{R}, Keio Collection, JW3778	(2)
1063	Δ*crp*	BW25113 Δ*crp*::*kan*^{R}, Keio Collection, JW3320	(2)
990	Δ*ptsI*	BW25113 *ptsI* gene deletion	(3)
979	Δ*crr*	BW25113 Δ*crp*::*kan*^{R}, Keio Collection, JW2410	(3)
1196	*crp**	BW25113 *crp* (A98T-C188A-A337C-C383T-G433A)	(4)
1066	Δ*cpdA*Δ*dosP*	BW25113 *cpdA* and *dosP* genes were deleted using CRISPR	This work
1943	SodA-GFP	BW25113 *gfp* inserted in front of the *sodA* stop codon	This work
1944	WT/SoxS-GFP	BW25113 contains the *soxS-gfp* fusion expression vector	This work

Plasmids	genotype	Source/reference
pCP20	$rep_{pSC101}{}^{ts}$*bla cat cI*857P_R	(5)
pTargetF	rep_{pBR322}*bla* pj23119-N20-sgRNA pMB1 $Spec^R$	(6)
pCas	$repA_{p101}{}^{ts}$sgRNA-pMBI p_{araBAD} exo bet gam Kan^R	(6)
pTargetF-FRT	pTargetF with an N20 sequence targeting the FRT	(3)
pAJR70	pACYC184 carries the *gfp* gene	(7)
soxS-gfp	*gfpmut3b*-expressing plasmid with native *soxS* promoter	(8, 9)

Table S6. Primers used in the study.

Primer Name	Sequence（5′--3′）	Usage
R-KO-check[a]	GCTTGCTGTCCATAAAACCG	Universal downstream primer for KO mutant identification
F-*mtlA*-KO-check	TTTTAGCGAAAATCGCCGCC	Upstream primer for Δ*mtlA* identification
F-*cmtA*-KO-check	TTTCCGGGGAATGACGAACC	Upstream primer for Δ*cmtA* identification
F-*mtlD*-KO-check	GGGGTTAATTGCCTGATGCG	Upstream primer for Δ*mtlD* identification
F-*hxpA*-KO-check	CCATTTTGACGCCGGAACTC	Upstream primer for Δ*hxpA* identification
F-*hxpB*-KO-check	AAAACAGCAATGCGTCCAGC	Upstream primer for Δ*hxpB* identification
sodA-HA-up-F	CATCTCCGACGAGATGAGTG	To amplify a fragment of the *sodA* gene containing the upstream homology arm
sodA-HA-up-R	GCTCCTCGCCCTTGCTCACCATTTTTTTCGCCGCAAAACG	
gfp-HA-md-F	CGTTTTGCGGCGAAAAAAATGGTGAGCAAGGGCGAGGAGC	To amplify a fragment of the *gfp* gene
gfp-HA-md-R	CATTGCAGCAGGCGGCAAATGATTACTTGTACAGCTCGTCCATGCCG	
sodA-HA-dn-F	CGGCATGGACGAGCTGTACAAGTAATCATTTGCCGCCTGCTGCAATG	To amplify *sodA* downstream homology arm fragment
sodA-HA-dn-R	TGCGTAATTCCTCAATGGAGC	
sodA-*gfp*-check-up	GAACGGAGCGTAAAAACAGGCT	Upstream primer for *sodA*-*gfp* identification
sodA-*gfp*-check-dn	AGAAGGTGTGGCACAGTGCG	Downstream primer for *sodA*-*gfp* identification
cpdA-sgRNA-F	GGACTAGTTGAGCGAGTTTCAGCTTGAGGTTTTAGAGCTAGAAATAGC	To amplify *cpdA*-specific sgRNA
cpdA-sgRNA-R	CTCAAAAAAAGCACCGACTCGG	
cpdA-HA-up-F	CCGAGTCGGTGCTTTTTTTGAGAGTCCGTCAGCAGTTCAAACG	To amplify *cdpA* upstream homology arm fragment
cpdA-HA-up-R	TGAAACCTAAGGACACCATTTGTGATGTCTACGCTTCTTTATTTA	
cpdA-HA-dn-F	TAAATAAAGAAGCGTAGACATCACAAATGGTGTCCTTAGGTTTCA	To amplify *cdpA* downstream homology arm fragment
cpdA-HA-dn-R	AACTGCAGTGGAATCGACCCGATACACTA	
cpdA-check-F	TTAAGATCGTAAATATGGCGTGA	Upstream primer for *cpdA* identification
cpdA-check-R	TCACAATTGCGCCGTTTGTTACC	Downstream primer for *cpdA* identification
dosP-sgRNA-F	GGACTAGTATCCAATTTGATCCGATGACGTTTTAGAGCTAGAAATAGC	To amplify *dosP*-specific sgRNA
dosP-sgRNA-R	CTCAAAAAAAGCACCGACTCGG	

Table S6. continued		
dosP-HA-up-F	CCGAGTCGGTGCTTTTTTGAGTCAGGGAAGATTACGGCTAAA	To amplify *dosP* upstream homology arm fragment
dosP-HA-up-R	CCAGGATGCAGAGGTAATCATGTGACAAATTCCTCTCGCCCG	
dosP-HA-dn-F	CGGGCGAGAGGAATTTGTCACATGATTACCTCTGCATCCTGG	To amplify *dosP* downstream homology arm fragment
dosP-HA-dn-R	AACTGCAGTGTTTTCCGCTACGGGGGCGA	
dosP-check-F	AGACAACGCGCTTGCTGATAAAT	Upstream primer for *dosP* identification
dosP-check-R	GCCATAACACTGGTGATGAAATT	Downstream primer for *dosP* identification
F-16S-RT	CTTACGACCAGGGCTACACAC	For RT-qPCR
R-16S-RT	CGGACTACGACGCACTTTATG	For RT-qPCR
F-*sucA*-RT	CTTCATCGACCTGGTGTGCT	For RT-qPCR
R-*sucA*-RT	GGTTAACCATCTCGGTGGCA	For RT-qPCR
F-*sucB*-RT	GGTGTGTTCGGTTCCCTGAT	For RT-qPCR
R-*sucB*-RT	TTCGCGACCATCGATCAGAC	For RT-qPCR
F-*sucC*-RT	ACCGGCTAACTTCCTTGACG	For RT-qPCR
R-*sucC*-RT	AACACCCACTTCTGCTACCG	For RT-qPCR
F-*mdh*-RT	GACCAAACGCATCCAGAACG	For RT-qPCR
R-*mdh*-RT	GTACTGACCGTCGCCTTCAA	For RT-qPCR
F-*nuoA*-RT	AAACGTGCCGTTTGAATCCG	For RT-qPCR
R-*nuoA*-RT	AGCTTCCACAAAGCCTACCC	For RT-qPCR
F-*nuoB*-RT	ACCGGTTATTCAGCGTCTGT	For RT-qPCR
R-*nuoB*-RT	CCTGCATGTACGCTTCAGGA	For RT-qPCR
F-*nuoC*-RT	GTGCTGGCGGTAGAGAAACT	For RT-qPCR
R-*nuoC*-RT	CTGACGATCGGTAAAGGCGA	For RT-qPCR
F-*sdhA*-RT	TTACCGTTGCGCTGGGTAAT	For RT-qPCR
R-*sdhA*-RT	CCATCATCGAGACGCGAGAA	For RT-qPCR
F-*sdhB*-RT	TCTGGCCTGTATTACCCCGA	For RT-qPCR
R-*sdhB*-RT	AGCTGGCGGATTTTGTCCAT	For RT-qPCR
F-*sdhC*-RT	TAGCGTCCATTCTCCATCGC	For RT-qPCR
R-*sdhC*-RT	GTGATACGCCAGAGCGGTAA	For RT-qPCR
F-*atpA*-RT	TATCGTGAACTGGCAGCGTT	For RT-qPCR
R-*atpA*-RT	CCAGGTAACCACGTTCTGCT	For RT-qPCR
F-*atpB*-RT	CGTTCAATCACTGGGCGTTC	For RT-qPCR
R-*atpB*-RT	CGGCACATTCAGGATCCACT	For RT-qPCR
F-*atpC*-RT	AAACAGCACGGTCACGAAGA	For RT-qPCR
R-*atpC*-RT	GCCGTGAGAGCTGCTAATGT	For RT-qPCR
F-*cyaA*-RT	TTTGCCAGCGAAGGGATCAT	For RT-qPCR
R-*cyaA*-RT	GCGATGACGAGTAGAAGCGA	For RT-qPCR

Table S6. continued		
F-*crp*-RT	CCCATCCAAGAGCACGCTTA	For RT-qPCR
R-*crp*-RT	AGGCCCAGTTCGCCAATAAA	For RT-qPCR

a: R-KO-check is a universal downstream primer paired with F-genes-KO-check, designed to be inside the kanamycin resistance gene. b: com-sgRNA-R F: forward; R: reverse

Supplementary References

1. Livak KJ, Schmittgen TD. 2001. Analysis of relative gene expression data using real-time quantitative PCR and the $2^{-\Delta\Delta C}$T Method. Methods 25:402-8.
2. Baba T, Ara T, Hasegawa M, Takai Y, Okumura Y, Baba M, Datsenko KA, Tomita M, Wanner BL, Mori H. 2006. Construction of *Escherichia coli* K-12 in-frame, single-gene knockout mutants: the Keio collection. Mol Syst Biol 2:2006.0008.
3. Zeng J, Hong Y, Zhao N, Liu Q, Zhu W, Xiao L, Wang W, Chen M, Hong S, Wu L, Xue Y, Wang D, Niu J, Drlica K, Zhao X. 2022. A broadly applicable, stress-mediated bacterial death pathway regulated by the phosphotransferase system (PTS) and the cAMP-Crp cascade. Proc Natl Acad Sci U S A 119:e2118566119.
4. Dessein A, Schwartz M, Ullmann A. 1978. Catabolite repression in *Escherichia coli* mutants lacking cyclic AMP. Mol Gen Genet 162:83-7.
5. Cherepanov PP, Wackernagel W. 1995. Gene disruption in *Escherichia coli*: TcR and KmR cassettes with the option of Flp-catalyzed excision of the antibiotic-resistance determinant. Gene 158:9-14.
6. Jiang Y, Chen B, Duan C, Sun B, Yang J, Yang S. 2015. Multigene editing in the *Escherichia coli* genome via the CRISPR-Cas9 system. Appl Environ Microbiol 81:2506-14.
7. Roe AJ, Yull H, Naylor SW, Woodward MJ, Smith DG, Gally DL. 2003. Heterogeneous surface expression of EspA translocon filaments by *Escherichia coli* O157:H7 is controlled at the posttranscriptional level. Infect Immun 71:5900-9.
8. Dwyer DJ, Kohanski MA, Hayete B, Collins JJ. 2007. Gyrase inhibitors induce an oxidative damage cellular death pathway in *Escherichia coli*. Mol Syst Biol 3:91.
9. Dwyer DJ, Belenky PA, Yang JH, MacDonald IC, Martell JD, Takahashi N, Chan CT, Lobritz MA, Braff D, Schwarz EG, Ye JD, Pati M, Vercruysse M, Ralifo PS, Allison KR, Khalil AS, Ting AY, Walker GC, Collins JJ. 2014. Antibiotics induce redox-related physiological alterations as part of their lethality. Proc Natl Acad Sci U S A 111:E2100-9.
